# Supplementary material for: Rapid turnover of life-cycle-related genes in the brown algae
Source: Genome Biol. 2019 Feb 14;20:35. doi: 10.1186/s13059-019-1630-6 (PMC6374913; doi:10.1186/s13059-019-1630-6)
Supplement: Supplementary file 3 — Protein alignments, corresponding Gblocks html file and CDS sequences for the lineage conserved gametophyte-biased genes. (ZIP 2109 kb) [file 13059_2019_1630_MOESM3_ESM.zip › Con_GA_alignments/Ec-19_002690.aa_ali.fasta-gb.htm]

Ec-19\_002690.aa.fasta.trans.aa\_ali.fasta


## Gblocks 0.91b Results

Processed file: **Ec-19\_002690.aa.fasta.trans.aa\_ali.fasta**  
Number of sequences: **2**  
Alignment assumed to be: **Protein**  
New number of positions: **414** (selected positions are underlined in blue)

```
                         10        20        30        40        50        60
                 =========+=========+=========+=========+=========+=========+
Ec-19_002690     MATLDRLARMDGLDVGEEDTDDGSIDQTIPSSSRPLFSLNEGIGNLRRQNNEPGNRPADV
MSTRG.25506.1    ------------------------------------------------------------
                                                                             


                         70        80        90       100       110       120
                 =========+=========+=========+=========+=========+=========+
Ec-19_002690     SFVDSDESMGTSARRQTQRADKLQPRDPTEDVLPEADLGDEPVHHEAKFNDDKNARAILT
MSTRG.25506.1    ------------------------------------------------------------
                                                                             


                        130       140       150       160       170       180
                 =========+=========+=========+=========+=========+=========+
Ec-19_002690     SGENGSGDTSRNTLDNLRNTFPVTRRAATPPPGYRPWGSSVSYSSSQRHPVDILERRGFV
MSTRG.25506.1    ------------------------------------------------------------
                                                                             


                        190       200       210       220       230       240
                 =========+=========+=========+=========+=========+=========+
Ec-19_002690     SNTRDTSHGTAKGDGANAADAASTITRLTVSLKRAQEEAARERERREVCEEQATAAYASL
MSTRG.25506.1    ------------------------------------------------------------
                                                                             


                        250       260       270       280       290       300
                 =========+=========+=========+=========+=========+=========+
Ec-19_002690     EAESKRACELDNRVRDADILLTRNGHGGRRGDNSGKGAAGGLFGGFSLNSHNRDRKTRSF
MSTRG.25506.1    ------------------------------------------------------------
                                                                             


                        310       320       330       340       350       360
                 =========+=========+=========+=========+=========+=========+
Ec-19_002690     SPDRNGASRVRAVEAAEETETHMAVIADATEKAREEAQSALDTVEAFAALNRKLVDELRT
MSTRG.25506.1    ------------------------------------------------------------
                                                                             


                        370       380       390       400       410       420
                 =========+=========+=========+=========+=========+=========+
Ec-19_002690     TVCARDELLGDLRNERGIREQLEHDIEELLGSGLVAEFKLREEDLKAQCKREQGESARVA
MSTRG.25506.1    ------------------------------------------------------------
                                                                             


                        430       440       450       460       470       480
                 =========+=========+=========+=========+=========+=========+
Ec-19_002690     KVHARMEEEYRIALDSSGRERARLQETVSSLEETLLSDRREHAISAREFSERATMGSEEM
MSTRG.25506.1    ------------------------------------------------------------
                                                                             


                        490       500       510       520       530       540
                 =========+=========+=========+=========+=========+=========+
Ec-19_002690     RSLRAGFLTRPRQRGYDRHTVRPSAAKLSGQGGSGGQGDVSGWLFDVQQRMDAQKVGAGR
MSTRG.25506.1    ------------------------------------------------------------
                                                                             


                        550       560       570       580       590       600
                 =========+=========+=========+=========+=========+=========+
Ec-19_002690     GVVRHPTKKTAPDGGRGVGHQNTGSDGNLSSAAVVPPTGGAKRQFTQPTSCGGGGGGGGD
MSTRG.25506.1    ------------------------------------------------------------
                                                                             


                        610       620       630       640       650       660
                 =========+=========+=========+=========+=========+=========+
Ec-19_002690     GSSIGIGGTGSGDGNAGQAVHAAAVENVELFLGDFVPEVRAQPQAASALLSASANASGSG
MSTRG.25506.1    ---FGNGGAGNGDGSLGPAVHASAVASVESFLGDFVPKVRAKPVAVSALLSAAVAPEEKG
                     ########################################################


                        670       680       690       700       710       720
                 =========+=========+=========+=========+=========+=========+
Ec-19_002690     -AEGKVGGEEHDGSAVVKKGSKWAAIGRGGKISARAAAVCLEEANKAAQAETLHRLRSMI
MSTRG.25506.1    SGDGRSGGD--DGGATEKKVSKWAAIGRAGKMSARATAVCAEEAIKAAEAEASHRLRSLI
                            #################################################


                        730       740       750       760       770       780
                 =========+=========+=========+=========+=========+=========+
Ec-19_002690     NAAARTLSASWFSLLATATSAAATH---GGHVGHGTGGGRGEGTAVENIN-------SAA
MSTRG.25506.1    NSAARALSASWFWLLTMVPATVAAARGTSTHGGHGGDGGRGDGVTEGSGNNNHVGGEGGG
                 #######################       ####################          


                        790       800       810       820       830       840
                 =========+=========+=========+=========+=========+=========+
Ec-19_002690     DRPASTDATVPAIAVRRLVQSCGILRNAGGGPTLAHADMELQRNTASRGGRSVGHREYFK
MSTRG.25506.1    KTSGEFSATVPAIVVRRLFQSCGILRSSAGGPTLAHADMELQRNAVGKGGRSVGHQEYFR
                        #####################################################


                        850       860       870       880       890       900
                 =========+=========+=========+=========+=========+=========+
Ec-19_002690     IIMALSQYRFVGDAKDHALEQLSRMLRPVQRDSPRTPPHAQGRQPVPRSAKLSAGDAAIA
MSTRG.25506.1    AVVALSHFRFAGESKDEALEKLSRMLHQAQPASAGTALHTRVRQREHQGPGHIAGSAHVV
                 ############################################                


                        910       920       930       940       950       960
                 =========+=========+=========+=========+=========+=========+
Ec-19_002690     NS-------------------V-PT---KGASLNEGLDGAERVLERDRRALEAMFLHYST
MSTRG.25506.1    EPLAAKPSPNGTSNITSVHVSTNPVHSMAGTGPEDGLAGAERVLERDRRALDAIFVHYST
                                              ###############################


                        970       980       990      1000      1010      1020
                 =========+=========+=========+=========+=========+=========+
Ec-19_002690     CGLLPFSALAAFCKDFELTPGLIPRDSAHRIYVQLAYPNEPALTPKLLAAREEPPGRRSA
MSTRG.25506.1    GGLVHSAALASFCKDFELIPGLISRDEVHRIYVQLAYPNETSLTPKLLAAREGRQGRR--
                 ##########################################################  


                       1030      1040      1050      1060      1070      1080
                 =========+=========+=========+=========+=========+=========+
Ec-19_002690     AAPATAARNQFGEGGVVAAAGLGLDQWMAWLGLVAVGCSWFDKQRGLYNTPTKKVLGLLQ
MSTRG.25506.1    -AGREAGRDEEGE-AAGGGGGLGVDQWITWLGLAAIGCSWFDKQRGLYNTPEKKVLGLLQ
                  ############       ########################################


                       1090      1100      1110
                 =========+=========+=========+======
Ec-19_002690     WMEGSVGKKRISAKRGGAFVPAFNLLPLKVDHRQRP
MSTRG.25506.1    WMEGSVGKKRISSKRGATIVPAFNLLPLRAM---RR
                 ############################
```

```
Parameters used
Minimum Number Of Sequences For A Conserved Position: 2
Minimum Number Of Sequences For A Flanking Position: 2
Maximum Number Of Contiguous Nonconserved Positions: 8
Minimum Length Of A Block: 10
Allowed Gap Positions: None
Use Similarity Matrices: Yes
```

```
Flank positions of the 7 selected block(s)
Flanks: [605  660]  [672  743]  [751  770]  [788  884]  [930  1018]  [1022  1033]  [1041  1108]  

New number of positions in Ec-19_002690.aa.fasta.trans.aa_ali.fasta-gb:  414  (37% of the original 1116 positions)
```
